# Wound drainage measurements: a narrative review

**DOI:** 10.1007/s00403-023-02525-5

**Published:** 2023-01-21

**Authors:** Terri Shih, Sarah Park, Linnea R. Thorlacius, Steven Daveluy, Amit Garg, Susanne D. Goegji, Joslyn S. Kirby, Barry M. McGrath, Peter T. Riis, Bente Villumsen, Kari Zalik, Gregor B. E. Jemec, Jennifer L. Hsiao

**Affiliations:** 1 David Geffen School of Medicine, University of California Los Angeles, Los Angeles, CA USA; 2 Department of Dermatology, Zealand University Hospital, Roskilde, Denmark; 3 Department of Dermato-Venereology & Wound Healing Centre, Bispebjerg Hospital, Copenhagen, Denmark; 4 Department of Dermatology, Wayne State University, Detroit, MI USA; 5 Department of Dermatology, Donald and Barbara Zucker School of Medicine at Hofstra/Northwell, New Hyde Park, NY USA; 6 Amsterdam, The Netherlands; 7 Department of Dermatology, Penn State Milton S. Hershey Medical Center, Hershey, PA USA; 8 HS Ireland, County Clare, Ireland; 9 Danish HS Patients’ Association, Copenhagen, Denmark; 10 Toronto, Canada; 11 Health Sciences Faculty, University of Copenhagen, Copenhagen, Denmark; 12 Department of Dermatology, University of Southern California, 1441 Eastlake Ave, Ezralow Tower, Suite 5301, Los Angeles, CA 90033-9174 USA

**Keywords:** Hidradenitis suppurativa, Wound, Drainage, Measure, Tool

## Abstract

Drainage from chronic wounds can significantly negatively impact a patient’s quality of life. Change in severity of wound drainage is an important measure of treatment efficacy for wounds. This study reviews existing tools used to assess wound drainage. Qualitative drainage tools are overall less burdensome, and however, differences in user interpretation may reduce inter-rater reliability. Quantitative drainage tools enable more reliable comparisons of drainage severity and treatment response between patients but sometimes require equipment to administer, increasing responder burden. Gaps in the current wound drainage measurement landscape are highlighted. Many of the existing scales have not been validated in robust studies. There is also a lack of validated global drainage measurement tools for patients with chronic inflammatory skin disorders with drainage, such as hidradenitis suppurativa or pyoderma gangrenosum. Development of a succinct drainage measurement tool for inflammatory skin diseases where drainage is a prominent symptom will improve monitoring of meaningful treatment response.

## Introduction

Chronic wounds represent the largest direct medical cost of all skin conditions, costing nearly 9 billion USD in the United States annually [1]. A patient’s quality of life (QOL) can be as profoundly impaired by chronic wounds as by heart and renal disease [1]. Existing, comprehensive wound assessments evaluate varying wound characteristics, including amount of drainage, pain between and during dressing changes, odor, itching, bleeding, and impact of the wound on QOL. Evaluation of change in wound drainage helps inform clinicians regarding treatment efficacy.

Many drainage assessments are currently tailored to assess post-surgical wounds. However, several other cutaneous conditions involve wound drainage, including inflammatory skin disorders such as hidradenitis suppurativa (HS) and pyoderma gangrenosum (PG), neoplastic diseases such as malignant tumors, and chronic ulcers, including vascular, inflammatory, and rheumatologic ulcers [1]. Although studies have examined the impact of drainage on patient QOL, there is a lack of validated, targeted tools to specifically measure drainage in patients with inflammatory skin disorders such as HS and PG [2]. The aim of this study is to review existing tools used to assess drainage and highlight the lack of and need for global drainage measures in inflammatory draining skin conditions.

## Qualitative wound drainage tools

### Assessment of drainage severity

Several measures have been created for patients to qualitatively assess drainage severity on categorical or numerical scales (Table 1). An example of a categorical scale is the Malignant Wound Assessment Tool (MWAT), a validated measure with domains including clinical wound features (i.e., wound location, classification, and edema) and physical, emotional, and social impact of the wound [3]. Regarding drainage, patients are asked to rate the severity as “dry, minimal, moderate, or heavy.” Numerical rating scales ask patients to rate drainage severity, often on a scale of 0–10. Zero represents no drainage, and “10” represents the worst drainage; “10” may be defined in different ways depending on the measure. As an example, the Toronto Symptom Assessment System for Wounds (TSAS-W) defines “10” as “most severe and/or continuous drainage or exudation.” [4] Some numerical scales have a descriptor that accompanies each number in the scale. Finally, another measure of drainage severity is qualitatively evaluating the amount of leakage from dressings. The Wound Symptoms Self-Assessment Chart queries how often fluid has been leaking from a patient’s dressing over the last week on a scale of 0–10, with “10” defined as “constantly leaking” [5]. Similarly, the Wound Management Questionnaire asks patients whether fluid has leaked through their dressing, from “not at all,” “a little,” “quite a bit,” to “a lot” [6].

**Table 1:** Qualitative wound drainage tools

Qualitative tools						
Name	Scale	Measurement of drainage	Other variables measured	Assessed by patient, caregiver, or HCP	Validated?	Example of study utilizing the scale
Drainage severity, assessed by patient						
Malignant Wound Assessment Tool—Clinical (MWAT) [3]		Dry Minimal Moderate Heavy	Clinical wound features, physical effects and emotional and social impacts of the wound	Patient	Yes	Breast wound [29]
Toronto Symptom Assessment System for Wounds (TSAS-W) [4]	0–100	0 = No drainage or exudation 10 = Most severe and/or continuous drainage or exudation	Odor, itching, bleeding, cosmetic concern, swelling/edema, bulk/mass effect from wound, bulk/mass effect from dressings	Patient or caregiver	No	Breast wound [30]
Wound Symptoms Self-Assessment Chart (WoSSAC) [5]	0–10	How often has fluid been leaking from your dressing over the last week? 0 = No fluid leaking 10 = Constantly leaking	Pain from wound, pain related to dressing changes, fluid leakage from dressing, bleeding, smell, itching	Patient	No	Acute and chronic wounds in a wound cleansing trial [31]
Wound Management Questionnaire (Elliott et al.) [6]		In the past 24 h, has fluid leaked through the dressing? Not at all, A little, Quite a bit, A lot	Dressing replacement in the last 24 h	Patient	No	Surgical sites [32]
Drainage severity, assessed by provider						
National Wound Assessment Form [7]		Wound moisture level Dry, Moist, Wet, Saturated, Leaking		HCP	No	NA
World Union of Wound Healing Societies’ Initiative Exudate Assessment (WUWHS) [8]		Dry Wound bed is dry; no visible moisture and primary dressing is unmarked; dressing may be adherent to wound Moist Small amounts of fluid are visible when the dressing is removed; primary dressing may be lightly marked; dressing change frequency appropriate for dressing type Wet Small amounts of fluid are visible when the dressing is removed; the primary dressing is extensively marked, but strikethrough is not occurring; dressing change frequency is appropriate for dressing type Saturated Primary dressing is wet, and strikethrough is occurring; dressing change is required more frequently than usual for the dressing type; peri-wound skin may be macerated Leaking Dressings are saturated and exudate is escaping from primary and secondary dressings onto clothes or beyond; dressing change is required much more frequently than usual for dressing type	Exudate color, consistency, odor (see “Drainage appearance” below)	HCP	No	NA; referenced in treatment consensus for epidermolysis bulla [33] and chronic wound management [34]
New Wound Bed Score (WBS) [9]	0–2	0 = Severe 1 = Moderate 2 = None/Mild	Edges, eschar, depth and granulation tissue, edema, dermatitis, peri-wound callus/fibrosis, wound bed	HCP	Yes	Lower extremity wounds [9]
Modified tissue, inflammation/infection, moisture, edge/epithelialization (TIME) [10]	0–2	0 = No exudate 1 = Exudate 2 = Smelly exudate	Age, mental state, self-sufficiency, nutrition, predisposing disease	HCP	Yes	NA
Pressure Ulcer Scale for Healing (PUSH tool) [11]	0–17	0 = None 1 = Light 2 = Moderate 3 = Heavy	Area (cm2), tissue type	HCP	Yes	Pressure ulcers [35]
Leg ulcer measurement tool [12]	0–56	0 = None 1 = Scant 2 = Small 3 = Moderate 4 = Copious	Type, size, undermining, necrotic tissue type, necrotic tissue, granulation tissue, skin viability, leg edema, assessment of bioburden	HCP	Yes	Lower extremity ulcers [36]
Drainage appearance						
Bates–Jenson Wound Assessment Tool (BWAT) [14]	1–60	Exudate type: 1 = Bloody (thin, bright red) 2 = Serosanguineous (thin, watery pale red to pink) 3 = Serous (thin, watery, clear) 4 = Purulent (thin or thick, opaque tan to yellow) 5 = Foul purulent (thick, opaque yellow to green with offensive odor)	Size, edges, undermining, necrotic tissue, skin color, peripheral tissue edema, induration, granulation, epithelialization, drainage amount (see Table 2)	HCP	Yes	Post-surgical healing [37]
World Union of Wound Healing Societies’ Initiative Exudate Assessment (WUWHS) [8]		Color: clear, amber; cloudy, milky or creamy; pink or red; green; yellow or brown; gray or blue Viscosity: high viscosity, low viscosity	Drainage volume (see “Drainage amount, assessed by provider” in Table 1)	HCP	No	See above
Change in drainage						
NCT03249909		Exudate status: Dry, Moist, Wet, Saturated, Leaking Change in exudate status: decrease, equal/unchanged, increase		HCP	No	NA

*HCP* Health care provider

Similarly, both categorical and numerical tools exist for healthcare providers (HCPs) to qualitatively assess drainage severity (Table 1). Categorical tools include the National Wound Assessment Form, which assesses wound moisture level as dry, moist, wet, saturated, or leaking [7]. The World Union of Wound Healing Societies’ (WUWHS) Initiative Exudate Assessment uses the same categorical rating scale for drainage, but provides definitions for each rating based on qualitative estimates of fluid amount, saturation of dressing, and frequency of dressing changes [8]. Several tools grade drainage on a numerical scale with a descriptor provided for each number. For most of these measures, “0” represents no drainage. Greatest severity is defined as “severe” in the New Wound Bed Score [9], “smelly exudate” in the Tissue, Inflammation/Infection, Moisture, Edge/Epithelialization (TIME) score [10], “heavy” in the Pressure Ulcer Scale for Healing tool [11], and “copious” in the Leg Ulcer Measurement Tool [12].

### Assessment of drainage appearance

In addition to assessing severity of drainage, certain tools also describe other characteristics of drainage, including viscosity, color, and the presence of odor. For example, MEASURE and MWAT can be used to evaluate drainage viscosity in categories ranging from serous, serosanguineous, to purulent [3, 13]. Drainage color is included in WUWHS, including options of red/pink, yellow/green, to brown/gray [8]. The presence of odor is a component of the TSASW and WUWHS measures [4, 8]. The Bates–Jenson Wound Assessment Tool (BWAT) scores exudate type on a scale of 1 (bloody) to 5 (foul purulent) based on a composite evaluation of viscosity, color, and the presence or absence of odor [14].

### Advantages and disadvantages of qualitative methods

Qualitative wound drainage measures do not require any equipment and are less burdensome to complete as compared to quantitative measures. Ease of use of these tools may encourage more frequent assessments of drainage, which is an important component of evaluating treatment efficacy for inflammatory skin conditions with drainage. Categorical and numerical scales for drainage amount can help track disease status and direction of change for individual patients. Disadvantages include the lack of specific criteria for each numerical or categorical value. Even for tools that define each possible response, user interpretations may vary since the definitions are not based on quantitative criteria, and this may reduce inter-rater reliability. Thus, comparing drainage status between patients based on qualitative responses is difficult (e.g., one patient’s “severe” may be another patient’s “moderate”). To this end, some clinical trials specifically track change in exudate status, as opposed to the exudate amount itself, noted as decreased, equal/unchanged, or increased [15].

## Quantitative wound drainage tools

### Assessment of drainage based on wound dressing

Several quantitative wound drainage tools use dressings as a means of drainage measurement (Table 2). Most are clinician-reported outcomes. Strategies include measuring the layers of gauze a wound soaks through, the number of dressing changes per day, and the degree of saturation on a dressing or on clothing. In the wound exudate score, users count the layers of gauze soaked through by exudate. The score ranges from 0 to 3, with 3 defined as > 12 layers of gauze soaked through [16]. Examples of measures that count the number of dressing changes include DESIGN-R and MEASURE. DESIGN-R evaluates frequency of dressing changes from 0 (none) to 6 (more than twice a day) [17]. MEASURE takes into consideration the wear time of a dressing in days and whether nonabsorptive or absorptive dressings are required [13]. Some clinical trials calculate the mean number of dressing changes per day and the total number of dressing changes needed until a wound has completely healed [18]. The Wound Management Questionnaire is a patient tool that asks patients whether leakage has required bedding or clothes to be changed, and if so, how many times [6].

**Table 2:** Quantitative wound drainage tools

Quantitative tools						
Name	Scale	Measurement of drainage	Other variables measured	Assessed by patient, caregiver, or HCP	Validated?	Example of study utilizing the scale
Number of dressing changes						
Wound exudate score [16]	0–3	0 = < 4 layers of gauze that the wound exudate wets through 1 = 4–7 layers of gauze that the wound exudate wets through 2 = 8–11 layers of gauze that the wound exudate wets through 3 = > 12 layers of gauze that the wound exudate wets through		HCP	No	NA
DESIGN-R assessment of progression toward healing [17]	0–28	0 = None 1 = Slight; does not require daily dressing change 3 = Moderate; requires daily dressing change 6 = Heavy; requires dressing change more than twice a day	Wound depth, size, inflammation/infection, granulation, necrosis, and pocket	HCP	Yes	Pressure ulcers [38]
MEASURE method [13]	0–3	0 = none (no exudate) 1 = small (fully controlled, ± nonabsorptive dressing, wear time ≤ 7 days) 2 = moderate (controlled, ± absorptive dressing, wear time 2–3 days) 3 = large (uncontrolled, absorptive dressings required, dressing may be overwhelmed in < 1 day)	Measure (size), appearance, suffering, undermining, wound edge	HCP	No	Post-surgical [39] and chronic wounds [40]
NCT02732886 [18]		Mean number of dressing changes per day Total number of dressing changes until complete wound healing		HCP	No	NA
Wound Management Questionnaire (Elliott et al.) [6]		Has the leakage required bedding or clothes to be changed? Yes (If “Yes” how many times?) No	Fluid leakage in the last 24 h	Patient	No	See above
Saturation through dressing/clothing						
Wound Fluid Quantification Score [19]		Absent = no moisture on gauze over 24 h Minimal = < 5 cc fluid on gauze over 24 h Moderate = 5–10 cc fluid on gauze over 24 h High = > 10 cc fluid on gauze over 24 h	Qualification of exudate (serious to purulent) and wound base (dry, granulating, ischemic, etc.)	HCP	No	NA
Treatment Evaluation by A Le Roux's Method (TELER) [20]	0–5	0 = dressing(s) and (bed)clothes are sodden 1 = dressing(s) and (bed)clothes are wet 2 = dressing(s) wet and (bed)clothes are damp 3 = dressing(s) wet and (bed)clothes are soiled in patches 4 = dressing(s) only is wet 5 = dressing(s) only is soiled		Patient or clinician	Yes	NA
Bates–Jenson Wound Assessment Tool (BWAT) [14]	1–60	1 = None (wound tissues dry) 2 = Scant (wound tissues moist, no measurable exudate) 3 = Small (wound tissues wet, drainage involves ≤ 25% dressing) 4 = Moderate (wound tissues saturated; involves 25%-75% of dressing) 5 = Large (wound tissues bathed in fluid, involves > 75% of dressing)	Size, edges, undermining, necrotic tissue, skin color, peripheral tissue edema, induration, granulation, epithelialization, drainage appearance (see Table 1)	HCP	Yes	See above
Stannard et al. wound grade criteria [21]	1–6	1 = No drainage 2 = Scant, no more than 3 small (< 4 mm) drops on removed dressing 3 = Minimal, 2 or less drops < 2 cm in size on removed dressing 4 = Mild, spots > 2 cm, not full length of incision on removed dressing 5 = Moderate, drainage along full length of incision on removed dressing 6 = Marked, soaks the dressing between changes		HCP	No	NA
Percent of wound affected						
Additional treatment, Serous discharge, Erythema, Purulent exudate, Separation of deep tissues, Isolation of bacteria, and Stay (ASEPSIS) Score [22]	0–40	0 = 0% wound affected 1 = < 20% wound affected 2 = 20–39% wound affected 3 = 40–59% wound affected 4 = 60–79% wound affected 5 = > 80% wound affected	Additional treatment, erythema, and separation of the deep tissues, the isolation of bacteria, and the duration of inpatient stay	HCP	Yes	Surgical wounds [41]
Volume of drainage						
NCT04656145 [23]		Total drainage: volume in cubic cm (via surgical drain)		HCP	No	NA
NCT03031314 [24]		Incision drainage in grams (dressing saturation size and weight)		HCP	No	NA
ESTimation method [25]		Wound cleansed, then covered with a transparent occlusive dressing. Fluid retained within the film was withdrawn using a micropipette. Collected samples were centrifuged, and volume of supernatant was measured using a micropipette in microliters, in units of ten. Volume per day was then calculated		HCP	No	NA
Skin humidity						
Kekonen et al. [26]		Assess degree of skin humidity via measurement of skin impedance using electrical circuits/electrodes to identify differences in electrical conductivity (low skin impedance is correlated with higher exudate production)		HCP	No	NA
Moisture monitor						
Henricson et al. [27]		Assess exudate levels via moisture sensor to determine when to change dressings for exudative wounds		HCP/Patient	No	NA

*HCP* Healthcare provider, *NA* not applicable

Different tools evaluate the degree of saturation on dressing or clothing. The Wound Fluid Quantification Score has an HCP rate drainage as absent, minimal, moderate, or high, based on the volume in milliliters of fluid on gauze over 24 h [19]. The Treatment Evaluation by A Le Roux's (TELER) Method evaluates whether dressings and/or clothes are damp, wet, or sodden [20]. BWAT examines the moisture of wound tissues (dry, moist, wet, saturated, or bathed in fluid) and area of drainage involvement of dressing (ranging from no measurable exudate to exudate involving > 75% of dressing) [14]. Stannard et al. quantify drainage via the size of exudate area involvement in centimeters on the removed dressing [21].

### Assessment of drainage based on amount of wound affected

One tool that assesses drainage severity based on the amount of wound affected is the validated Additional treatment, Serous discharge, Erythema, Purulent exudate, Separation of deep tissues, Isolation of bacteria, and Stay (ASEPSIS) Score (Table 2). Utilizing a numerical scale of 0 to 5, it examines serous and purulent exudate separately and defines each score based on proportion of the wound affected (i.e., a score of 5 indicates > 80% wound affected) [22].

### Other quantitative measures of drainage

Some clinical trials seek to measure the exact volume of drainage. These methods may include measurement in cubic cm of drainage collected in surgical drains [23] or calculating the weight difference of a saturated dressing [24]. The ESTimation method uses a micropipette to recover fluid from a dressing that was occluded over the wound. The sample is centrifuged to remove cell debris, and then, the volume of supernatant is measured in microliters. The volume per hour is then multiplied by 24 to calculate drainage over 24 h [25].

Recent studies have examined whether skin impedance values, which reflect the response of skin regions to applied electrical currents, may help characterize drainage. Kekonen et al. described the utility of using electrodes to generate skin impedance values to assess degree of skin humidity [26]. Low skin impedance was correlated with higher exudate production. Henricson et al. described using a moisture sensor to assess exudate levels to guide when to change wound dressings [27].

### Advantages and disadvantages of quantitative methods

Quantitative tools for wound drainage severity measurement have less inter-rater variability as compared to qualitative methods. They not only enable the evaluation of drainage status and drainage response to treatment of individual patients, but also allow more reliable comparisons regarding drainage severity and treatment response across different patients. However, quantitative methods impart greater responder burden on the clinician and patient, sometimes requiring physical equipment. In addition, unless exact measurement of drainage is sought, surrogate methods of measurement may still impart inter-rater variability. For example, measures based on number of dressing changes per day may differ depending on different thresholds patients have for changing their dressing. Measures that examine dressing saturation depend on how the dressing is affixed and the type of dressing used. Of the measures discussed in this paper, only DESIGN-R, TELER, and BWAT have been validated in studies.

### Lack of global drainage measures in chronic inflammatory skin conditions

Using HS as an example of a chronic inflammatory draining skin disease, there is one study in the literature that evaluates efficacy of wound dressings in HS patients, which asked participants to rate drainage severity from 1 to 4, defined as 1 (no), 2 (a little), 3 (moderate), or 4 (a lot of drainage) [28]; however, this was not a validated measure. Another measure, the Hidradenitis Suppurativa Odor and Drainage Scale (HODS), asks patients to rate both the usual amount and worst amount of drainage from their HS for specific anatomic locations, including head/neck, armpits, trunk, groin, buttocks, genital/perianal area, and other areas. The 5-point categorical scale for HODS ranges from “no drainage” to “very severe drainage,” and each is defined based on the wear time of dressings. Though specific for HS, HODS does not include a simple global drainage measure. PG, another chronic draining inflammatory skin condition, also lacks a specific global measure for drainage.

## Discussion

Draining skin lesions greatly influence the morbidity of patients and constitute a significant clinical problem. However, the clinimetrics of drainage are overall poorly developed. Although there are various qualitative and quantitative wound drainage measurements that are in use as clinical tools or have been used in clinical trials, many of the existing scales have not been validated in robust studies.

There is currently a lack of a global, validated tool assessing wound drainage in inflammatory skin disorders. Several of the existing wound drainage measures are designed for surgical wounds and pressure ulcers, and these measures are not readily applicable to inflammatory skin conditions. For instance, measuring the distribution of drainage in a wound bed is difficult in a disease like HS where drainage is often sequestered in tunnels. Characterizing drainage viscosity as a measure of drainage severity is also less relevant for non-infectious, inflammatory skin conditions.

The lack of a specific and validated global drainage tool for inflammatory skin diseases is a barrier to assessment of this important outcome. More studies should investigate the development of a succinct drainage measure in skin diseases where drainage is a prominent, burdensome symptom. This would allow for improved monitoring of meaningful treatment response in clinical trials as well as in clinical settings.
